# A Case Report of Brachiocephalic Vein Spasm Secondary to Peripherally Inserted Central Catheter

**DOI:** 10.7759/cureus.27037

**Published:** 2022-07-19

**Authors:** Seth Russell, Jordan H Chamberlin, Jeremy R Burt, Ismail M Kabakus

**Affiliations:** 1 Diagnostic Radiology, Medical University of South Carolina, Charleston, USA; 2 Cardiothoracic Imaging, Medical University of South Carolina, Charleston, USA

**Keywords:** venous spasm, venospasm, brachiocephalic vein spasm, deep vein thrombosis, central catheter insertion

## Abstract

Vascular spasm is well known and studied in the arterial system. There are only a few cases reported related to central venous spasms. We present the case of a 63-year-old male with an extensive medical history, including deep vein thrombosis (DVT), who underwent peripheral insertion of a central catheter in his left upper extremity with subsequent development of left upper extremity edema. The central catheter was removed before the patient underwent a contrast-enhanced computed tomography of the chest which revealed severe narrowing of the left brachiocephalic vein, consistent with venospasm in the clinical setting. Nitroglycerin might be useful to prevent vasospasm, or it might also be used for treatment. In our case, the catheter was removed, and no subsequent treatment was necessary.

## Introduction

Although large-caliber venous spasms have been reported, they are rare and less studied compared to arterial spasms. The few similar cases of large venous spasm are associated with the percutaneous intervention, including inferior vena cava spasm, left axillary vein spasm, and left subclavian vein spasm [1-3]. To our knowledge, this is the first case report of venospasm of the brachiocephalic vein. This contrasts with coronary artery spasms associated with the percutaneous coronary intervention, a similar phenomenon with more robust evidence for causation [4].

There is evidence, however, with a small sample size, that pre-procedural intravenous administration of 200 μg nitroglycerin can reduce the severity of axillary venospasm during pacemaker placement [5]. There are also two case reports of attempting to treat axillary venospasm after venous puncture using intravenous (IV) nitroglycerin through the ipsilateral vein; however, both were unsuccessful [3,6]. The limited data suggest that IV nitroglycerin may successfully prevent venous spasm when administered through the ipsilateral vein pre-procedurally; however, it has not been a successful treatment in the few case reports of IV administration after the venospasm has occurred.

## Case presentation

**Figure 1:**
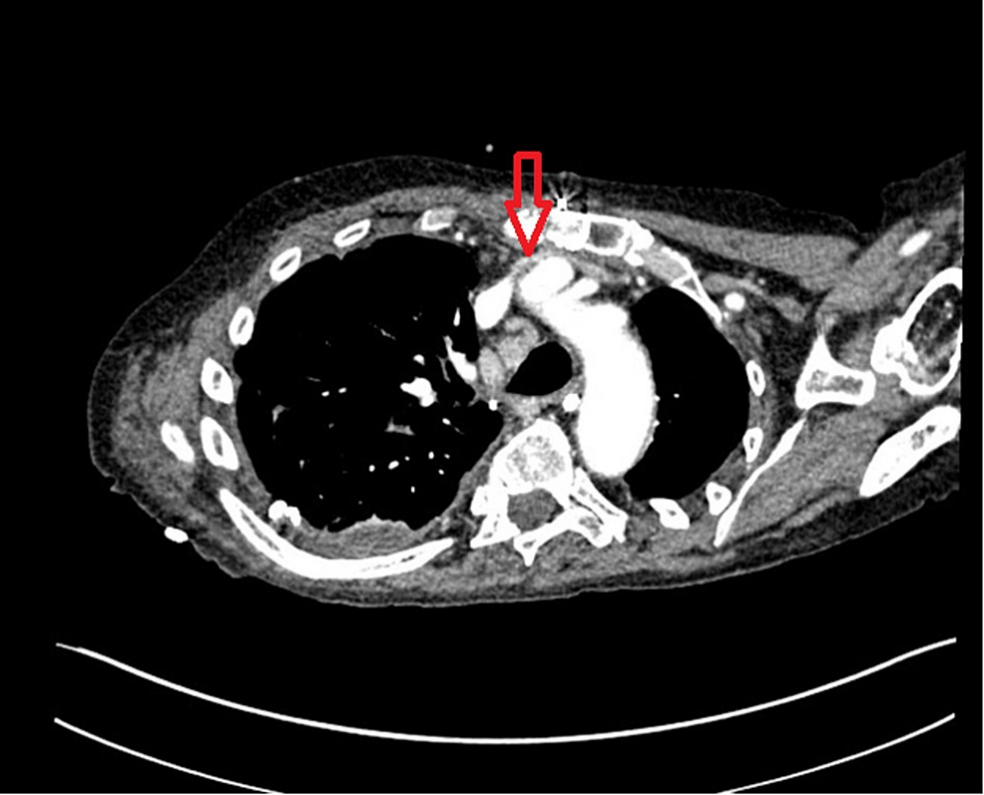
Chest CT scan with contrast done the day of catheter placement showing severe narrowing of the left brachiocephalic vein. CT: computed tomography

**Figure 2:**
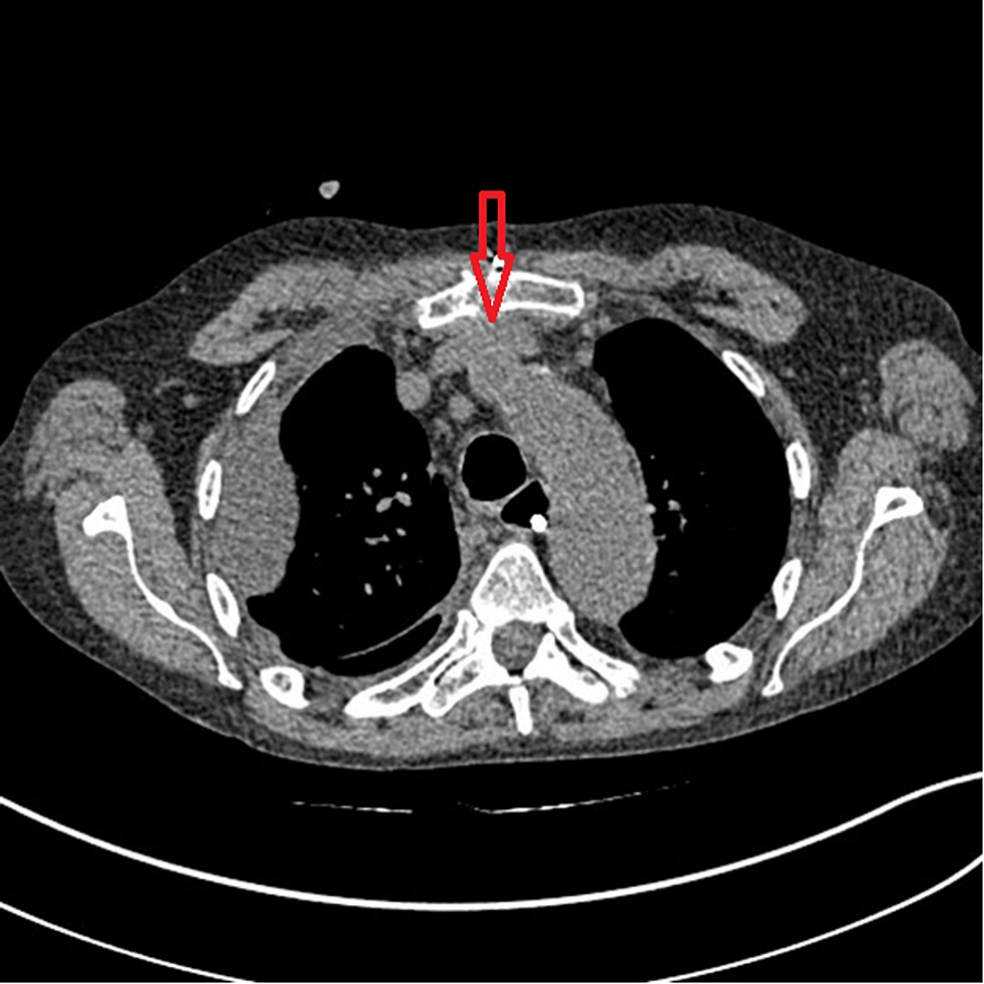
Non-contrast chest CT scan performed 10 days prior to venospasm demonstrating a normal-caliber brachiocephalic vein with no evidence of focal narrowing. CT: computed tomography

**Figure 3:**
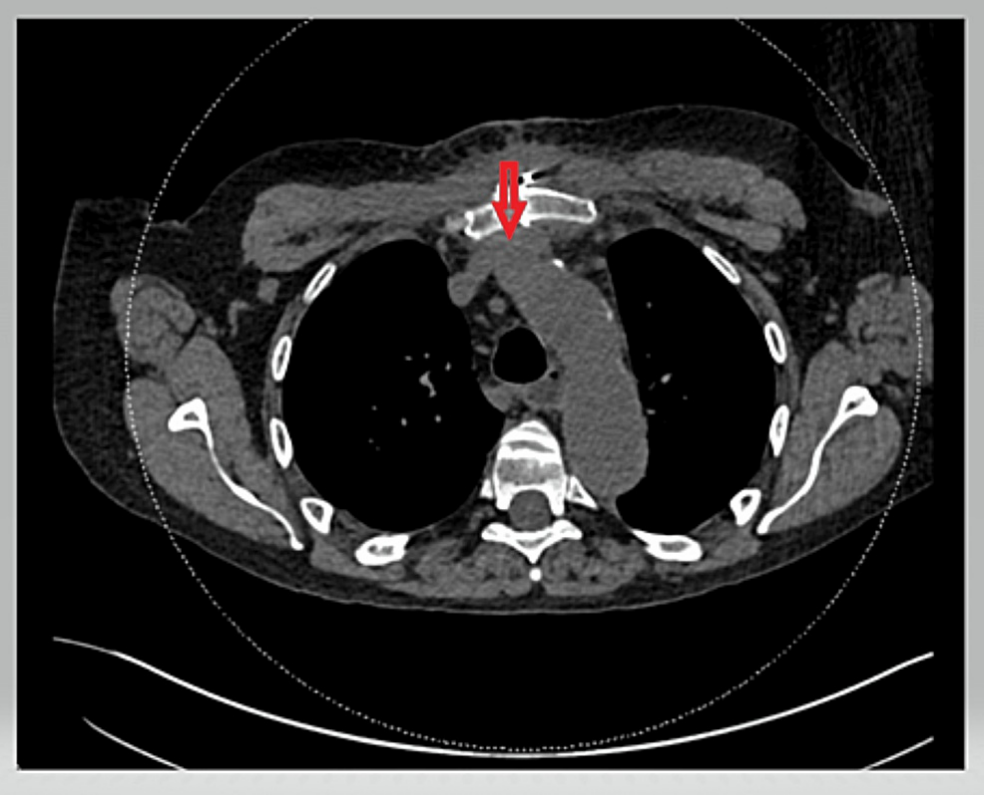
Non-contrast chest CT performed two months later showing the left brachiocephalic vein had returned to its previous caliber without stenosis. CT: computed tomography

We present the case of a 63-year-old male with an extensive medical history, including deep vein thrombosis (DVT). A peripherally inserted central catheter (PICC) line was inserted in the left brachial vein and advanced centrally without immediate adverse events. A few hours later, the patient developed left upper extremity pitting edema. At this point, the PICC line was removed, and a contrasted chest computed tomography (CT) was performed for suspicion of venous thrombosis, considering the patient's previous vein thrombosis history. The chest CT revealed severe narrowing of the left brachiocephalic vein just posterior to the sternum without evidence of a filling defect. The narrowing of the brachiocephalic vein can be seen in Figure 1. There was no stenosis or narrowing seen on comparison chest CT performed 10 days prior (Figure 2), giving a diagnosis of central catheter-induced brachiocephalic vein spasm. Administration of nitroglycerin was a consideration, but, in this case, no additional treatment was necessary, and the edema was relieved within the following one to two hours. Figure 3 shows a non-contrast CT performed two months later showing that the brachiocephalic vein had returned to its normal caliber without stenosis.

## Discussion

The physiology of vascular vasospasm is not fully understood. The most established and accepted mechanisms are endothelial dysfunction, nitric oxide release, and enhanced vascular smooth muscle cell contractility as disruptions of vascular tone [4,7]. However, endothelial damage due to physical manipulation by a catheter has been suggested as a cause of vasospasm in coronary and cerebral angiography [8,9]. In fact, Ishihara et al. found that both larger catheter size and catheter contact with the vessel wall were significantly associated with the incidence of coronary arterial vasospasm, supporting the role of mechanical trauma in vasospasm pathophysiology [8]. It has also been proposed that vascular smooth muscle cell contraction is exacerbated by a superimposed primary nonspecific hyperreactivity, the mechanisms of which have not been fully elucidated [9]. Regardless, the literature on venospasm is sparse and the literature on the physiology of mechanical venospasm is incomplete.

In this study, we report that large vessel venospasm occurred after instrumentation of the brachiocephalic vein, adding to the body of evidence suggesting endothelial dysfunction due to physical is likely to be a risk factor for vasospasm. Clinicians should be aware of the risk for central venospasm after PICC line insertion, a condition that may mimic DVT, another common adverse event associated with PICC placement. The difference is considerable as anticoagulation would not be indicated for a patient with vasospasm. The key CT finding of acute vein thrombosis is normal or slightly enlarged vessel diameter with a luminal filling defect. However, severe narrowing of the vessel lumen with the unimpeded flow in a patient with relevant history is a sign of venospasm.

## Conclusions

Central venous spasm can occur after PICC line insertion, a condition that may mimic acute DVT. This case report adds to a small but growing body of literature that instrumentation of large vessel veins can cause venospasm. It is important to differentiate venous spasm from vein thrombosis as anticoagulation would not be indicated for a patient with vasospasm. Radiologists should recognize the severe narrowing of the vasculature with the unimpeded flow in a patient with a central line as a sign of venospasm.
